# Development and evaluation of a community-based buprenorphine treatment intervention

**DOI:** 10.1186/s12954-017-0149-y

**Published:** 2017-05-12

**Authors:** Aaron D. Fox, Nancy L. Sohler, Taeko Frost, Carolina Lopez, Chinazo O. Cunningham

**Affiliations:** 1 Albert Einstein College of Medicine, Bronx, NY 10461 USA; 2 Montefiore Medical Center, 111 E. 210th Street, Bronx, NY 10467 USA; 3 City College of New York, Sophie Davis School of Biomedical Education, New York, NY 10031 USA; 4 Washington Heights CORNER Project, New York, NY 10033 USA; 5 New York Harm Reduction Educators, New York, NY 10035 USA

**Keywords:** Buprenorphine, Harm reduction agencies, Access to care, Opioid addiction

## Abstract

**Background:**

The majority of Americans with opioid use disorder remain out of treatment. Operating in 33 states, Washington DC, and Puerto Rico, harm reduction agencies, which provide sterile syringes and other health services to people who inject drugs, are a key venue to reach out-of-treatment opioid users. Aiming to link out-of-treatment individuals with opioid use disorder to buprenorphine treatment, we developed a community-based buprenorphine treatment (CBBT) intervention in collaboration with New York City harm reduction agencies.

**Methods:**

Intervention development included formative data collection, feasibility testing at one harm reduction agency, and pilot testing for preliminary effectiveness at a second harm reduction agency. We used a pre-post design for both feasibility and pilot testing. In the CBBT intervention, we trained harm reduction agency staff to provide (1) buprenorphine education, (2) motivational interviewing, (3) referrals to buprenorphine-prescribing doctors, and (4) treatment retention support. We assessed feasibility by measuring staff satisfaction with the intervention and changes in knowledge about buprenorphine. We assessed preliminary effectiveness by comparing rates of buprenorphine initiation among groups of harm reduction agency clients before and after intervention implementation.

**Results:**

Among staff members at the first harm reduction agency, knowledge increased from 52% correct answers pre-intervention to 79% correct post-intervention. Among clients at the second harm reduction agency, initiation of buprenorphine treatment was low and did not differ between pre- and post-intervention groups.

**Conclusions:**

The CBBT intervention was feasible and well-received, but initiation of buprenorphine treatment among harm reduction agency clients was low. More robust interventions may be necessary to increase initiation of buprenorphine treatment.

## Background

The USA is in the midst of an “epidemic” of opioid use, disorder, and overdose, yet the majority of opioid users remain out of treatment [1, 2]. The traditional infrastructure of drug treatment programs only reaches a fraction of those who may need treatment. In 2014, approximately 586,000 persons met criteria for heroin use disorder, and more than 1.9 million met criteria for prescription opioid use disorder [3]; however, admissions to treatment facilities for opioid use disorder were only 471,000—less than 20% of those needing treatment—leaving a large treatment gap [4]. The Drug Addiction Treatment Act of 2000 has allowed for office-based buprenorphine treatment as an alternative to drug treatment programs [5]; however, the treatment gap has persisted over the past decade, suggesting that more effort is needed to engage out-of-treatment opioid users [6].

The reasons why opioid users do not enter drug treatment are complex. Costs, availability, and stigma of drug treatment may be barriers [3, 7, 8]. Office-based buprenorphine treatment is safe and effective, covered by Medicaid in many states, and may be less stigmatized than drug treatment programs, but opioid users still must actively seek out treatment [9–13]. Though data on patient-level barriers to buprenorphine treatment are lacking, informational barriers, or lack of knowledge of available treatment options, may play an important role [14].

Harm reduction agencies serve a diverse population of out-of-treatment opioid users, many of whom report interest in buprenorphine treatment [15]. In the USA, harm reduction agencies are the main source of sterile syringe exchange for injection drug users. Many agencies also provide case management, group and individual mental health services, testing for human immunodeficiency virus (HIV) and hepatitis A, B, or C virus, overdose prevention training, and a variety of peer-delivered services [16]. In 2013, there were 204 known syringe exchange programs in 33 states, Washington DC, and Puerto Rico, a slight increase from 184 programs in 2009 [17, 18]. More than 80% of syringe exchange participants report injecting heroin [18]. Therefore, syringe exchange programs and harm reduction agencies are a key target for outreach to opioid users in need of treatment. A few pilot programs have implemented buprenorphine treatment within harm reduction agencies, but this venue has not been fully realized as a means to engage an out-of-treatment population in buprenorphine treatment [19–21].

With the goal of linking out-of-treatment opioid users to buprenorphine treatment, we developed a community-based buprenorphine treatment (CBBT) intervention in collaboration with harm reduction agencies in New York City. Clients of harm reduction agencies face multiple barriers to addiction treatment [22]; therefore, we undertook a comprehensive intervention development approach to identify and address potential barriers. Here, we describe the conception, development (including evaluation for feasibility), and pilot testing of the CBBT intervention to assess preliminary effectiveness.

## Methods

The CBBT intervention development began with formative data collection that guided intervention design and implementation. We developed, evaluated, and refined the intervention in collaboration with one harm reduction agency (feasibility). We then pilot tested the CBBT intervention at a second harm reduction agency, examining whether clients exposed to the intervention initiated buprenorphine treatment (preliminary effectiveness). The study was approved by affiliated Institutional Review Boards.

### Preliminary needs assessment

Participants at harm reduction agencies were directly involved in development of the CBBT intervention. We surveyed clients of several harm reduction agencies in New York City to establish experience with, interest in, acceptability of, and barriers to buprenorphine treatment. In 2009, most clients were aware of buprenorphine, many had acquaintances who received buprenorphine treatment, but very few had been prescribed buprenorphine [23]. We conducted six focus groups with 38 harm reduction agency clients to better understand their attitudes toward and access to buprenorphine treatment [24]. This study confirmed high levels of interest in buprenorphine and the demand for accurate information regarding buprenorphine treatment. Participants perceived that they were unable to access buprenorphine treatment in their community, which led them to use illicit buprenorphine for “self-treatment” of opioid addiction. However, participants also reported willingness to access services at harm reduction agencies and identified staff at harm reduction agencies as key sources of information and services. We then surveyed an additional 162 harm reduction agency clients, which confirmed positive attitudes toward buprenorphine treatment with 86% agreeing that buprenorphine reduced opioid use and 71% agreeing that those taking buprenorphine were ready to change their addiction-related behaviors [25]. Therefore, as described below, we conceived of the CBBT intervention to meet the demand for accurate information regarding buprenorphine treatment and improved access to care.

### Setting

With the goal of training harm reduction agency staff to provide education and facilitate referrals to buprenorphine treatment among their clients, we collaborated with two harm reduction agencies—one to develop the CBBT intervention and one to pilot test it. Development occurred with the New York Harm Reduction Educators (NYHRE), which is the largest harm reduction agency in New York City with over 35 staff members and serving over 5000 clients annually. At two community-based offices and 11 street-side locations, NYHRE provides participants with syringe exchange; referral for medical, dental, and addiction treatment; acupuncture; HIV risk reduction education and interventions; HIV case management; and mental health services. The majority of NYHRE’s clients are Hispanic or black, male, 40–49 years old, and injection drug users. NYHRE leadership identified staff members who were most likely to engage out-of-treatment opioid users (e.g., case managers, outreach workers, and syringe exchange program staff), and we developed procedures to train them in the CBBT intervention.

Pilot testing of the CBBT intervention occurred with the Washington Heights CORNER Project (WHCP), which is another harm reduction agency situated in a community highly affected by opioid addiction and HIV. At its office, WHCP provides clients with sterile syringes; case management; referrals for medical, dental, or addiction treatment; HIV risk reduction education and interventions; and harm reduction counseling. WHCP serves more than 1500 clients, the majority of whom are Hispanic or Black, male, 40–49 years old, and inject drugs. WHCP employs 19 staff members. WHCP leadership identified staff members who were most likely to engage out-of-treatment opioid users, and we trained them in the CBBT intervention.

### Developing and evaluating the CBBT intervention

Based on formative focus group and survey data, we designed the CBBT intervention to include four components that were aimed at staff of harm reduction agencies. We targeted staff and not clients so that education about and referrals to buprenorphine treatment would become integrated into harm reduction agency procedures instead of relying on external research staff. These four components were (1) buprenorphine education for staff, with the goal of informing harm reduction clients about buprenorphine treatment and dispelling commonly held myths; (2) motivational interviewing training for staff, with the goal of helping clients develop strategies to change behaviors related to opioid use, including initiating buprenorphine treatment; (3) training staff to identify buprenorphine-prescribing doctors and facilitate clients’ linkage to care; and (4) training staff to support clients receiving buprenorphine treatment to improve treatment retention. Staff trainings occurred between September 2010 and May 2012 among 22 unique staff members. We evaluated the CBBT intervention components by assessing staff satisfaction with each component and change in staff knowledge with the education component.

In the Table 1, we provide a detailed description of the content, delivery, and evaluation of the four CBBT intervention components (feasibility).

**Table 1:** Development and evaluation of the community-based buprenorphine treatment (CBBT) intervention components

Method	Content description	Evaluation
Component 1: Buprenorphine Education^a^		
Slide presentation	8 modules: introduction; background information on buprenorphine; what is buprenorphine; who can take buprenorphine; getting on buprenorphine; buprenorphine and the brain; buprenorphine/naloxone vs. buprenorphine alone; effectiveness and safety of buprenorphine; being involved in your buprenorphine treatment	Mean satisfaction^b^ = 1.9 Pre-post knowledge increased from 50 to 79% of answers correct.^c^
Frequently asked questions	30 questions frequently asked by people seeking buprenorphine treatment	
Resource guide	Resources (websites and phone numbers) that provide buprenorphine information and list buprenorphine-prescribing physicians	
Glossary of terms	42 frequently used terms associated with opioid addiction and buprenorphine	
Myths and facts about buprenorphine	16 commonly held myths regarding buprenorphine, and the facts regarding these statements	
Component 2: Motivational Interviewing^d^		
Slide presentation	Components: what is motivational interviewing; stages of change; key principles of motivational interviewing	Mean satisfaction^e^ = 4.5
Workbook	Self-reflection activities, stages of change clinical scenarios, role-playing activities	
Component 3: Facilitate access to buprenorphine-prescribing physicians		
Identification of buprenorphine-prescribing physicians	Interactive exercises using websites to locate buprenorphine-prescribing physicians	Mean satisfaction^b^ = 1.6
Facilitate clients’ accessing buprenorphine treatment	Role play phone calls to doctors’ offices inquiring about buprenorphine treatment; exploration of process to initiate buprenorphine treatment; role play potential structural barriers (insurance issues, medication cost, transportation)	
Component 4: Support during buprenorphine treatment		
Case scenarios with common treatment challenges	Group discussion of 4 case scenarios that represent common treatment challenges: use of illicit buprenorphine; relapse; polysubstance use; severe untreated mental illness.	Mean satisfaction^b^ = 1.6

^a^Modified from the New York City Department of Health and Mental Hygiene’s Buprenorphine Educational Curriculum

^b^Satisfaction was measured using an 8-item questionnaire that rated importance, usefulness, comfort, and appropriateness of the trainings’ content and structure. Ratings used a Likert scale from 1 to 4, where 1 = highly satisfied and 4 = highly dissatisfied

^c^Knowledge was measured before and after the training via a 5-item multiple choice questionnaire

^d^Developed and delivered by the Harm Reduction Coalition [44]

^e^Satisfaction was measured using a Likert scale from 1 to 5, where 1 = poor and 5 = excellent

### Pilot testing of the CBBT intervention

After development was complete, to pilot test the preliminary effectiveness of the CBBT intervention, we repeated trainings with staff at a second harm reduction agency. Trainings were conducted with staff, but outcomes were assessed for clients of the harm reduction agency. Using a pre-post design, we compared buprenorphine initiation between two groups of harm reduction agency clients—those enrolled before implementing the CBBT intervention (pre-intervention group) and those enrolled after implementing the CBBT intervention (post-intervention group).

#### Participants

Participants were recruited via flyers posted at the harm reduction agency’s drop-in center and by staff referral. Participant eligibility criteria included (1) age >18 years, (2) fluency in English or Spanish, (3) client of the harm reduction agency, (4) history of injection drug use, (5) no prescribed buprenorphine in the previous 6 months, (6) self-report of having a problem with heroin or prescription opioids, and (7) interest in changing opioid use. The last two criteria were assessed via modified questions from a validated instrument [26]. Participants were ineligible if they were (1) pregnant or (2) taking over 60 mg of methadone in a treatment program because these criteria would make them clinically ineligible for buprenorphine treatment at the community health center to which they would be referred.

#### Study visits and data sources

We conducted three study visits with participants (at baseline, 30 days, and 60 days) and collected interviews and urine samples. Interviews were conducted using Audio Computer-Assisted Self-Interview (ACASI) technology in a private space in the harm reduction agency’s office by trained research staff. Interview data included sociodemographic characteristics, substance use [27], history of drug treatment, and HIV risk behaviors [28]. Urine collection was unobserved but urine temperature was assessed to determine validity of urine samples. Research staff tested urine samples for opiates, oxycodone, methadone, buprenorphine, cocaine, and benzodiazepines using Rapid TOX® (American Bio Medica, Kinderhook, NY).

#### Outcomes

The primary outcome was the number of participants initiating buprenorphine treatment during the 60-day follow-up period. Secondary outcomes included substance use (by self-report and urine drug testing) and HIV risk behaviors.

#### Delivery of the CBBT intervention

Once we reached our target sample of 50 pre-intervention participants and completed their follow-up interviews, we then delivered the CBBT intervention to the harm reduction agency staff. CBBT intervention components were delivered via weekly sessions over a 5-week period. After completion of the CBBT intervention, due to time constraints, we were unable to reach our goal of recruiting 50 post-intervention participants, but we did enroll 26. Of all the 76 participants, 66 (87%) had at least one follow-up interview.

#### Analysis

To determine whether the post-intervention group was more likely to initiate buprenorphine treatment than the pre-intervention group, we conducted chi-square tests comparing the pre- and post-intervention groups. We used similar methods to examine differences between groups for all secondary outcomes.

## Results

Of the 141 participants screened, 85 (60%) were eligible, and of these, 76 (89%) enrolled in the study. Among all participants, the mean age was 44.9 years, and the majority were non-Hispanic white (62%), high school graduates (76%), unemployed (83%), homeless (61%), had histories of incarceration (82%), and had public insurance (70%). Most (51%) reported infection with hepatitis C virus. Polysubstance use within the prior 30 days was common (99% reported using opioids, 68% cocaine, and 38% sedatives). In addition, 80% reported currently injecting drugs, 43% ever overdosing, 45% ever taking methadone, 9% currently receiving methadone treatment (dose of 60 mg or less), and 57% ever taking prescribed or illicit buprenorphine. There were no significant differences in these characteristics between the pre-and post-intervention groups.

### Primary outcome—initiation of buprenorphine treatment

Of the 50 pre-intervention participants, 2 (4%) initiated buprenorphine treatment over the follow-up period. Of the 26 post-intervention participants, none initiated buprenorphine treatment over the follow-up period.

### Secondary outcomes—opioid use and HIV risk behaviors

Opioid use (by self-report or urine toxicology testing) decreased in both the pre- and post-intervention groups over the follow-up period. In the pre-intervention group, opioid use was reported among 92% of participants at baseline and 83% of participants at follow-up. In the post-intervention group, opioid use was reported among 100% at baseline and 80% at follow-up. In the pre-intervention group, urine toxicology tests were positive for opiates in 80% at baseline and 73% at follow-up. In the post-intervention group, urine toxicology tests were positive for opiates in 83% at baseline and 68% at follow-up. Drug-related HIV risk behaviors only changed in a small fraction of participants. In the pre-intervention group, 6% increased high risk behaviors and 24% decreased high risk behaviors over the follow-up period. In the post-intervention group, 15% increased high-risk behaviors and 15% decreased high-risk behaviors over the follow-up period. None of these changes in drug use (by self-report or urine toxicology testing) or drug-related HIV risk behaviors were significantly different between pre- and post-intervention groups.

## Discussion

In collaboration with harm reduction agencies, we rigorously evaluated barriers to buprenorphine treatment among out-of-treatment opioids users and developed a community-based buprenorphine treatment (CBBT) intervention to overcome these barriers. Our goal was to provide a practical intervention that could easily be incorporated by harm reduction agencies. Training staff to provide buprenorphine education and referrals to buprenorphine treatment appeared to be feasible; however, initiation of buprenorphine treatment was low among harm reduction agency clients before and after the CBBT intervention.

Prior studies of harm reduction agencies (or syringe exchange programs) as conduits to addiction treatment have demonstrated both challenges and successes. Clients of harm reduction agencies express high levels of interest in addiction treatment, and epidemiologic studies have demonstrated an association between syringe exchange use and entrance into addiction treatment [23, 29, 30]; however, early efforts to refer syringe exchange participants to methadone maintenance or other addiction treatment resulted in only 5–10% entering treatment [31, 32]. A harm reduction agency in New Haven previously reported higher rates of referral and initiation of addiction treatment among clients, but few of those requesting referral utilized syringe exchange, and many sought treatment for substance use other than opioids [33]. An intervention in Baltimore combining motivational enhancement counseling and monetary incentives resulted in 40% of syringe exchange participants entering methadone maintenance treatment [34], which is consistent with the rate of treatment entrance among out-of-treatment injection drug users recruited from Denver streets when they were offered free treatment (including methadone maintenance) [35]. Thus, monetary incentives and free treatment may be a better approach to increasing treatment initiation than staff trainings. Some opioid users prefer buprenorphine treatment to methadone maintenance [36]; therefore, similar incentives for referral to buprenorphine treatment may be warranted.

Our inability to increase initiation of buprenorphine treatment with the CBBT intervention could stem from multiple sources. First, though interest in stopping opioid use was an inclusion criterion for the pilot study, addiction treatment may have been a lower priority for clients than other immediate needs (e.g., housing assistance). Therefore, staff may have been appropriately focused on clients’ higher priority needs instead of referrals to buprenorphine treatment. Second, harm reduction agencies are often grant funded, and competing priorities, such as service delivery and reporting to funders, may have made it difficult for staff to focus on referrals for buprenorphine treatment. An alternative approach with a dedicated buprenorphine referral coordinator may have been more effective. Third, the buprenorphine education component of the CBBT intervention appeared to be effective, but additional training in motivational interviewing may be necessary to help staff encourage harm reduction clients to initiate buprenorphine treatment. Recruiting from a harm reduction agency with syringe exchange is likely to select for a population more ambivalent about stopping opioids than a traditional treatment-seeking population. Increasing the time dedicated to training in motivational interviewing and assessing the quality of counseling delivered will be important for further evaluation of the CBBT intervention. Fourth, referrals were to a single community health center, which was not in the same neighborhood as the harm reduction agency. Proximity and availability of public transportation are important factors that could provide a barrier to buprenorphine treatment and will be important to consider when developing patient-centered models of treatment.

The strengths of our study include a thorough needs assessment, pre-testing of intervention components, and pilot testing in a sample of out-of-treatment opioid users who were not exposed to intervention development. We have developed training approaches and materials that increased buprenorphine knowledge and were well received by staff. Though rates of buprenorphine initiation were low among harm reduction clients, utilizing these materials in other settings (e.g., training care coordinators to facilitate referrals to buprenorphine treatment for health plan members) may facilitate access to buprenorphine treatment for other opioid users. Our collaboration with harm reduction agency leadership and staff was also fruitful and could serve as a model for academic and community partnerships. We have published several reports on the attitudes toward and access to buprenorphine treatment among harm reduction clients, and the perspectives of opioid users are needed to inform policy and enhance acceptability of treatment models [16, 23–25, 37].

Our next step in intervention development will be to include buprenorphine initiation at harm reduction agencies as part of the CBBT intervention. Rapid intake has repeatedly been shown to foster entrance into addiction treatment and ideally should happen on the same day of referral [38–40]. Additionally, opioid users may be reluctant to enter traditional medical settings when referred due to stigma and fear of discrimination [41]. We have already demonstrated that harm reduction clients would prefer to receive buprenorphine treatment onsite at harm reduction agencies rather than referral to drug treatment programs or medical clinics [16]. Onsite treatment could both prevent treatment delays and reduce stigma. Avoiding the need for referral to specialty clinics by integrating hepatitis C virus (HCV) treatment into primary care or addiction treatment settings has been a successful strategy to increase uptake of HCV treatment, and this experience can be applied to buprenorphine treatment [42, 43]. Reaching out-of-treatment opioid users will require models of care delivery that offer treatment in settings that are accessible and where they feel comfortable.

There are several limitations to our study. We were not able to randomize at the level of harm reduction agencies or clients, and the comparison of pre-intervention and post-intervention groups does not account for potential secular trends in utilization of buprenorphine treatment. Additionally, we were unable to reach our recruitment goal in the post-intervention group, so our negative findings may be due to lack of power. Our experience in New York City may not be generalizable to other geographic areas, and the clients of collaborating harm reduction agencies were mostly middle age; therefore, findings may not be generalizable to younger opioid users. We were unable to observe staff members delivering the CBBT intervention during the pilot test, so the fidelity of the intervention provided by staff members is unknown. Finally, urine drug testing was unobserved and testing the temperature of urine may have been insufficient to prevent tampering of urine samples.

## Conclusions

Harm reduction agencies have been critical in addressing the intertwined epidemics of opioid addiction and HIV infection and will continue to provide valuable services that improve the health of people who use drugs. Training harm reduction agency staff in the CBBT intervention was not effective at increasing initiation of buprenorphine treatment among opioid users, but trainings and materials were feasible and well received. While intervening at the level of harm reduction agency staff would have aided sustainability, alternative interventions, such as targeting clients with incentives for referral or offering buprenorphine treatment onsite at harm reduction agencies, could be evaluated to encourage initiation of buprenorphine treatment.

## Abbreviations

ACASI: Audio Computer Assisted Self-Interview
CBBT: Community-based buprenorphine treatment
HIV: Human immunodeficiency virus
NYHRE: New York Harm Reducation Educators
WHCP: Washington Heights CORNER Project
